# Supplementary material for: Prevalence and distribution of multilocus sequence types of Staphylococcus aureus isolated from bulk tank milk and cows with mastitis in Pennsylvania
Source: PLoS One. 2021 Mar 12;16(3):e0248528. doi: 10.1371/journal.pone.0248528 (PMC7954355; doi:10.1371/journal.pone.0248528)
Supplement: S1 Table — (DOCX) [file pone.0248528.s001.docx]

**S1 Table. List of primers and control strains used in the study**

| **Gene** | **Control Strain** | **Primer** | **Sequence** | **Size** | **Tm**  **(°C)^a^** | **Source** |
| --- | --- | --- | --- | --- | --- | --- |
| Carbamate kinase (arcC) | NA | arc F | 5' TTG ATT CAC CAG CGC GTA TTG TC -3' | ~500 | 55 | http://saureus.beta.mlst.net/ |
|  |  | arc R | 5' AGG TAT CTG CTT CAA TCA GCG -3' |  |  |  |
| Shikimate dehydrogenase (aroE) | NA | aro F | 5' ATC GGA AAT CCT ATT TCA CAT TC -3' | ~500 | 55 | http://saureus.beta.mlst.net/ |
|  |  | aro R | 5' GGT GTT GTA TTA ATA ACG ATA TC -3' |  |  |  |
| Glycerol kinase (glpF) | NA | glp F | 5' CTA GGA ACT GCA ATC TTA ATC C -3' | ~500 | 55 | http://saureus.beta.mlst.net/ |
|  |  | glp R | 5' TGG TAA AAT CGC ATG TCC AAT TC -3' |  |  |  |
| Guanylate kinase (gmk) | NA | gmk F | 5' ATC GTT TTA TCG GGA CCA TC -3' | ~500 | 55 | http://saureus.beta.mlst.net/ |
|  |  | gmk R | 5' TCA TTA ACT ACA ACG TAA TCG TA -3' |  |  |  |
| Phosphate acetyltransferase (pta) | NA | pta F | 5' GTT AAA ATC GTA TTA CCT GAA GG -3' | ~500 | 55 | http://saureus.beta.mlst.net/ |
|  |  | pta R | 5' GAC CCT TTT GTT GAA AAG CTT AA -3' |  |  |  |
| Triosephosphate isomerase (tpi) | NA | tpi F | 5' TCG TTC ATT CTG AAC GTC GTG AA -3' | ~500 | 55 | http://saureus.beta.mlst.net/ |
|  |  | tpi R | 5' TTT GCA CCT TCT AAC AAT TGT AC -3' |  |  |  |
| Acetyl coenzyme A acetyltransferase (yqiL) | NA | yqi F | 5' CAG CAT ACA GGA CAC CTA TTG GC -3' | ~500 | 55 | http://saureus.beta.mlst.net/ |
|  |  | yqi R | 5' CGT TGA GGA ATC GAT ACT GGA AC -3' |  |  |  |
| Staphylococcal enterotoxin A (sea) | ATCC 13565 | SEA F | 5' CCT TTG GAA ACG GTT AAA ACG -3' | 127 | 57 **^a^** | 24 |
|  |  | SEA R | 5' TCT GAA CCT TCC CAT CAA AAA C -3' |  |  |  |
| Staphylococcal enterotoxin B (seb) | ATCC 14458 | SEB F | 5' TCG CAT CAA ACT GAC AAA CG -3' | 477 | 57 **^a^** | 24 |
|  |  | SEB R | 5' GCA GGT ACT CTA TAA GTG CCT GC -3' |  |  |  |
| Staphylococcal enterotoxin C (sec) | ATCC 19095 | SEC F | 5' CTC AAG AAC TAG ACA TAA AAG CTA GG -3' | 271 | 57 **^a^** | 24 |
|  |  | SEC R | 5' TCA AAA TCG GAT TAA CAT TAT CC -3' |  |  |  |
| Staphylococcal enterotoxin D (sed) | ATCC 23235 | SED F | 5' CTA GTT TGG TAA TAT CTC CTT TAA ACG -3' | 319 | 57 **^a^** | 24 |
|  |  | SED R | 5' TTA ATG CTA TAT CTT ATA GGG TAA ACA TC -3' |  |  |  |
| Staphylococcal enterotoxin E (see) | ATCC 27664 | SEE F | 5' CAG TAC CTA TAG ATA AAG TTA AAA CAA GC -3' | 178 | 57 **^a^** | 24 |
|  |  | SEE R | 5' TAA CTT ACC GTG GAC CCT TC -3' |  |  |  |
| Staphylococcal enterotoxin G (seg) | ATCC 19095, ATCC 23235 | SEG F | 5' AAG TAG ACA TTT TTG GCG TTC C -3' | 287 | 57 **^a^** | 24 |
|  |  | SEG R | 5' AGA ACC ATC AAA CTC GTA TAG C -3' |  |  |  |
| Staphylococcal enterotoxin H (seh) | ATCC 19095 | SEH F | 5' GTC TAT ATG GAG GTA CAA CAC T -3' | 213 | 57 **^a^** | 24 |
|  |  | SEH R | 5' GAC CTT TAC TTA TTT CGC TGT C -3' |  |  |  |
| Staphylococcal enterotoxin I (sei) | ATCC 19095, ATCC 23235 | SEI F | 5' GGT GAT ATT GGT GTA GGT AAC -3' | 454 | 57 **^a^** | 24 |
|  |  | SEI R | 5' ATC CAT ATT CTT TGC CTT TAC CAG -3' |  |  |  |
| Staphylococcal enterotoxin like J (selj) | ATCC 13565, ATCC 23235 | SEJ F | 5' ATA GCA TCA GAA CTG TTG TTC CG -3' | 152 | 57 **^a^** | 24 |
|  |  | SEJ R | 5' CTT TCT GAA TTT TAC CAC CAA AGG -3' |  |  |  |
| Staphylococcal enterotoxin like K (selk) | ATCC 14458 | SEK F | 5' TAG GTG TCT CTA ATA ATG CCA -3' | 293 | 57 **^a^** | 24 |
|  |  | SEK R | 5' TAG ATA TTC GTT AGT AGC TG -3' |  |  |  |
| Staphylococcal enterotoxin like L (sell) | ATCC 19095 | SEL F | 5' TAA CGG CGA TGT AGG TCC AGG -3' | 383 | 57 **^a^** | 24 |
|  |  | SEL R | 5' CAT CTA TTT CTT GTG CGG TAA C -3' |  |  |  |
| Staphylococcal enterotoxin like M (selm) | ATCC 29213 | SEM F | 5' GGA TAA TTC GAC AGT AAC AG -3' | 379 | 57 **^a^** | 24 |
|  |  | SEM R | 5' TCC TGC ATT AAA TCC AGA AC -3' |  |  |  |
| Staphylococcal enterotoxin like N (seln) | MRSA CDC-1 | SEN F | 5' TAT GTT AAT GCT GAA GTA GAC -3' | 282 | 57 **^a^** | 24 |
|  |  | SEN R | 5' ATT TCC AAA ATA CAG TCC ATA -3' |  |  |  |
| Staphylococcal enterotoxin like O (selo) | ATCC 29213 | SEO F | 5' TGT GTA AGA AGT CAA GTG TAG -3' | 214 | 57 **^a^** | 24 |
|  |  | SEO R | 5' TCT TTA GAA ATC GCT GAT GA -3' |  |  |  |
| Staphylococcal enterotoxin like P (selp) | NA | SEP F | 5' TGA TTT ATT AGT AGA CCT TGG -3' | 396 | 57 **^a^** | 24 |
|  |  | SEP R | 5' ATA ACC AAC CGA ATC ACC AG -3' |  |  |  |
| Staphylococcal enterotoxin like Q (selq) | ATCC 14458, ATCC 27664 | SEQ F | 5' AAT CTC TGG GTC AAT GGT AAG C -3' | 122 | 57 **^a^** | 24 |
|  |  | SEQ R | 5' TTG TAT TCG TTT TGT AGG TAT TTT CG -3' |  |  |  |
| Staphylococcal enterotoxin like R (selr) | ATCC 13565 | SER F | 5' GGA TAA AGC GGT AAT AGC AG -3' | 166 | 57 **^a^** | 24 |
|  |  | SER R | 5' GTA TTC CAA ACA CAT CTA AC -3' |  |  |  |
| Toxic shock syndrome toxin (TSST1) | ATCC 51651 | TST F | 5' AAG CCC TTT GTT GCT TGC G -3' | 447 | 57 **^a^** | 24 |
|  |  | TST R | 5' ATC GAA CTT TGG CCC ATA CTT T -3' |  |  |  |
|  |  | FemB R | 5' ATA CAA ATC CAG CAC GCT CT -3' |  |  |  |
| Leukocidin (component M and F) (LukMF') | Isolate from bovine milk sample | LukMF F | 5' AAC TTT CAA TGA TGT TAA ACA AAA TAG AG -3' | 405 | 56 **^a^** | 27 |
|  |  | LukMF R | 5' AAA ATA GTC TCT AGC ATT AGG TCC -3' |  |  |  |
| Leukocidin (component E and D) (LukED) | ATCC 12600 | LukED F | 5' TGA AAA AGG TTC AAA GTT GAT ACG AG -3' | 269 | 60 | 26 |
|  |  | LukED R | 5' TGT ATT CGA TAG CAA AAG CAG TGC A -3' |  |  |  |
| Leukocidin (component A and B) (LukAB) | ATCC 12600 | LukAB F | 5' GCT CTA GAT AAG CTC ACA CCT TTT CAA AGT AGT -3' | ~410 | 60 | 25 |
|  |  | LukAB R | 5' GGG GTA CCA TGA TTG ATA TTT GTT GAT ATG -3' |  |  |  |

***^a^*** Primers used in the same multiplex PCR reaction.
