# Supplementary material for: Prevalence and distribution of multilocus sequence types of Staphylococcus aureus isolated from bulk tank milk and cows with mastitis in Pennsylvania
Source: PLoS One. 2021 Mar 12;16(3):e0248528. doi: 10.1371/journal.pone.0248528 (PMC7954355; doi:10.1371/journal.pone.0248528)
Supplement: S2 Table — (DOCX) [file pone.0248528.s002.docx]

**S2 Table. Clonal complexes and sequences types of *S. aureus* reported in literature.**

| **Country** | **Date** | **Source** | **MLST CCs and STs ^a^** | **Reference** |
| --- | --- | --- | --- | --- |
| Algeria | 2015 | Bovine mastitis and caretakers | **CC1,** CC5, CC25, CC22, **CC97** | 37 |
| Australia | 2012-2013 | Bovine milk | **CCI (ST1)**, CC5(**ST15**), CC8(ST8), **CC97**(ST3183, ST3184), **CC705(ST705)** | 2 |
| Brazil | 2001-2004 | Bovine milk | **CC1(ST1,** ST743) CC5(ST744), CC30(ST30), CC126(ST126, ST741), CC133(ST745) and **CC97(ST97**, ST742, ST746, ST747) | 82 |
| Brazil | - | Clinical mastitis | **CC1(ST1),** CC30, **CC97**(ST126) | 40 |
| Canada | 1958 | Clinical | **CC97**(ST115) | 16 |
| Canada | 2007-2008 | Clinical mastitis, milk samples | CC8 (ST8), **CC97(ST352, ST2187),** CC126(ST126, ST2270), CC133(ST133), **CC151 (ST151**, ST351**)** | 83 |
| Chile | - | Milk samples | **ST97,** ST355, ST356, ST357, ST358, ST359 | 20 |
| China | 2014-2015 | Mastitis | **CC1(ST1**, ST1920, ST81), CC5(ST6, ST965), CC7(ST7), CC20(ST20), CC25(ST25), CC40(ST50), CC50(ST59, ST537), CC88 (ST88), **CC97(ST97**), **CC151(ST151)**, CC188(ST188), CC520(ST520), **CC398(ST398),** CC630(ST630) | 51 |
| China | 2014 |  | **CC1**, CC5, **CC97** and CC188 | 12 |
| China | 2014-2015 |  | **CC1,** CC8, **CC45**, **CC97**, CC188, **CC398** | 65 |
| China | 2015 | Clinical mastitis | **CC1(ST1),** CC7(ST7), CC50(ST50), **CC97(ST97)**, **CC398(ST398)** | 41 |
| China | 2018 | Clinical mastitis | **CC1**(ST63, ST188, ST584, ST9, ST805, ST2139, ST1, ST2700), CC8(ST903, ST2373, ST2454), ST2990, ST968. | 84 |
| Denmark | 2016 | Clinical mastitis and bulk tank milk | **ST1,** ST7, ST8, ST9**, ST15,** ST50, ST71, **ST97,** ST132, ST133**, ST151, ST398,** ST479, ST504, ST705, ST706, ST1380, ST2423, ST3891, ST3892, ST3896, ST3897, ST3898, ST3899, ST3900,  ST4361, ST4362, ST4363, ST4364, ST4365, | 42 |
| Ethiopia | 2014-2016 | Milk samples | **CC1(ST1**, ST848), CC22(ST22), **CCT97**(ST4550), ST88 | 43 |
| France | 1961-2004 | Subclinical and clinical mastitis | **CC97(ST97,** ST71, **ST352),** CC20(ST389), ST1276 | 16 |
| Germany | 2006 | Subclinical mastitis | **CC97**(ST115), CC705(ST1274, ST504), CC479 (ST479), CC133(ST133), ST1275 | 16 |
| Germany | - | Bovine milk | **CC45, CC151**, CC479 | 44 |
| Germany | 2001-2014 | Milk samples | CC5(ST2825), CC8(ST7), CC50(ST2837) **CC97(CC97**, ST71, ST464, ST2824, ST2826), CC133(ST133, ST2821), **CC151(ST151**, ST504, ST2823), CC479(ST479, ST1380), CC398(ST398) | 52 |
| Iran | 2009-2017 | Bovine nasal swabs and milk | CC5(ST6) , **CC15(ST15),** ST291, **CC45(ST45).** | 45 |
| India | - | Milk samples | **CC1**(ST63), CC8(**ST72**, ST239), **CC97**(ST2459), ST88, ST1687 | 46 |
| Ireland | 1993 | Clinical mastitis | CC705(**ST151**) | 16 |
| Ireland | 2010-2011 | Clinical mastitis | CC136(ST136), **CC97(**ST71, ST3170), **CC151(ST151)** | 17 |
| Italy | 2011-2012 | Cows with high somatic cell counts | CC5, CC8(ST8), **CC97**, CC126 | 9 |
| Italy | 2012-2013 | Bulk tank milk | CC5(ST1490, ST2081, ST5), CC7(ST789), CC8(ST1652, ST3079, ST450, ST8), **CC15**, CC59(ST59), CC72(ST72), **CC97**(ST3077, ST9, **ST97**), CC126(ST126), CC133(ST30800, **CC151**(**ST151**, ST504), CC353(ST3084, ST71), CC389(ST389), CC479(ST479), CC522(ST522) | 47 |
| Japan | 1998-2005 | Bovine milk | CC5(ST5), CC6(ST1362, ST6), CC7(ST789), CC8(ST8, ST72, ST630), CC12(ST12, ST1369), **CC15**(**ST1**, ST81, ST188), CC20(ST20,ST1368, ST1370, CC25(ST25, ST26, ST1372), CC30(ST243), **CC45** (ST508), CC59(ST59), CC88(ST88, ST1360, **CC97(ST97,** ST124, **ST352**, ST1366), CC509(ST89), CC705(**ST705**, ST1363, ST1364, ST1365, ST1359, ST1361, ST1371) | 15 |
| Norway | 2005 | bulk tank milk | **CC1(ST1**, ST147**),** CC5(ST135), ST25, ST130, ST131, ST132, ST133, ST136, S478, ST479, ST481, ST483, ST520. | 48 |
| Rwanda | 2018 | Bovine mastitis | **CC97,** CC152, CC3591, CC3666, ST5475, ST5476, ST5477. | 85 |
| South Africa |  | Bovine milk and caretakers | CC8 (ST8), **CC45**( ST508), **CC97 (ST97, ST352**, ST2992, ST3538), CC705(ST351) | 13 |
| Switzerland | 2007-2009 | Bovine milk and caretakers | **CC1**, CC7, **CC15**, CC20, **CC45**, **CC97**, CC101, **CC151,** CC188 | 39 |
| Switzerland | 2011-2012 | Bovine mastitis | CC7, CC8, CC9, CC20, **CC45,** **CC97**, **CC151**,CC479. | 86 |
| Switzerland | 2017-2018 | Milk samples | **CC1 ( ST1),** CC8 (ST8), CC20 (ST389), **CC97 (ST71, ST352),** CC479 (ST479, ST1380), CC705 (**ST151**, ST504) | 38 |
| Thailand | 2016 | Subclinical bovine mastitis | **CC97**(ST1179), ST8, ST10, ST243, ST294, ST1123, ST1223, ST2250, ST2793, ST3879, ST3882, and ST3883 | 87 |
| Tunisia | 2013-2014 | Clinical mastitis | CC5(ST4118,ST4119), CC130(ST4115), **CC97**(ST4112, ST4113, ST4120), **CC151**(ST4117) | 88 |
| United Kingdom | - | Milk samples | ST9, **ST151** | 20 |
| United Kingdom | - | Bovine mastitis | **CC1(ST1), CC97(ST97**), ST130(ST130), **ST151(ST151),** ST188(ST188), CC133(ST771) | 49 |
| United States | 1988 | Milk samples | **ST1**, ST25. ST30, **ST45**, ST50, **ST97,** ST122, ST124, ST125, **ST151**, ST347, ST348, ST359, **ST350**, ST351, **ST352**, ST353, ST354 | 20 |
| United States | 2013 | Milk samples | CC5 (ST8), **CC15**(ST25), CC20(ST20), CC58(ST87), **CC97(ST352, ST2187,** ST 2189**),** CC705 (**ST151,** ST2185). | 50 |
| United States | 2010-2013 | Bulk tank milk Samples | **CC1(ST1),** CC8(ST8), CC30(ST30), **CC45**(ST3023), CC59(ST87), **CC97(ST97, ST352, ST693, ST2187,** ST3020, ST3021, ST3022, ST3024, **ST3028), CC151(ST151,** ST3026, ST3027), CC705**(ST705),** ND(350) | 32 |

*^a^*  Clonal Complex (CC) and Sequence Type (ST) in bold were also observed in this study; ND, Not Defined.
